# Potential sources of bacteria colonizing the cryoconite of an Alpine glacier

**DOI:** 10.1371/journal.pone.0174786

**Published:** 2017-03-30

**Authors:** Andrea Franzetti, Federico Navarra, Ilario Tagliaferri, Isabella Gandolfi, Giuseppina Bestetti, Umberto Minora, Roberto Sergio Azzoni, Guglielmina Diolaiuti, Claudio Smiraglia, Roberto Ambrosini

**Affiliations:** 1 Dept. of Earth and Environmental Sciences (DISAT) - University of Milano-Bicocca, Milano, Italy; 2 “A. Desio” Dept. of Earth Sciences, Università degli Studi di Milano, Milano, Italy; Free University of Bozen/Bolzano, ITALY

## Abstract

We investigated the potential contribution of ice-marginal environments to the microbial communities of cryoconite holes, small depressions filled with meltwater that form on the surface of Forni Glacier (Italian Alps). Cryoconite holes are considered the most biologically active environments on glaciers. Bacteria can colonize these environments by short-range transport from ice-marginal environments or by long-range transport from distant areas. We used high throughput DNA sequencing to identify Operational Taxonomic Units (OTUs) present in cryoconite holes and three ice-marginal environments, the moraines, the glacier forefield, and a large (> 3 m high) ice-cored dirt cone occurring on the glacier surface. Bacterial communities of cryoconite holes were different from those of ice-marginal environments and hosted fewer OTUs. However, a network analysis revealed that the cryoconite holes shared more OTUs with the moraines and the dirt cone than with the glacier forefield. Ice-marginal environments may therefore act as sources of bacteria for cryoconite holes, but differences in environmental conditions limit the number of bacterial strains that may survive in them. At the same time, cryoconite holes host a few OTUs that were not found in any ice-marginal environment we sampled, thus suggesting that some bacterial populations are positively selected by the specific environmental conditions of the cryoconite holes.

## Introduction

Glaciers and ice sheets represent the largest part of the cryosphere on the continents [1,2] and store most of the Earth’s freshwater. Cryoconite holes are small depressions on the ablation zone of glacier surfaces filled with water, whose formation is due to a thin layer of supraglacial debris (cryoconite). The dark cryoconite melts the underlying ice when heated by solar radiation [3] and forms a depression that can be filled by meltwater. Cryoconite holes range in diameter from a few centimetres to more than a meter, can cover up to 10% of the ablation zone of glaciers and can be considered autonomous micro-ecosystems [2,4], inhabited by many Archaea, bacteria, cyanobacteria, protists and micro-invertebrates [4,5]. They are also considered the most biologically active environments on the glaciers due to the high metabolic versatility of their biological communities [6]. Primary productivity can be surprisingly high in these extreme micro-ecosystems, and can support simple, stable trophic webs that can sustain secondary consumers and predators, such as tardigrades, rotifers, nematodes or copepods [5,7,8]. Importantly, recent studies demonstrated that microbial growth in cryoconite increases the amount of dark-coloured organic matter and significantly reduces the ice albedo, thus increasing glacier melting rate [9].

Diversity, functions and assembly processes of microbial communities in cryoconite have been investigated both on Arctic and Antarctic glaciers [10–13] and on temperate mountain glaciers [14–22]. These studies highlighted that, on three high-Arctic glaciers, cryoconite and ice-marginal environments host distinct communities, and only a minority of bacterial phylotypes occurred in both environments [23]. This difference can be because cryoconite holes offer different ecological niches for bacteria from those of ice-marginal environments, mainly due to the presence of melted water [14]. Not surprisingly, their fauna was more similar to that of the surrounding aquatic ecosystems than to that of surrounding soil habitats [8] and it has been hypothesized that presence of supraglacial lakes may influence bacterial communities of cryoconite holes [24]. However, on temperate mountain glaciers with no large supraglacial lakes, almost all water ecosystems disappear from the glacier surface during winter, including cryoconite holes themselves, which can therefore be considered ephemeral environments on these glaciers [25,26]. This is a main difference between cryoconite holes on temperate mountain and Polar glaciers, where cryoconite holes can persist for several ablation seasons and are therefore relatively stable environments [5].

Since on temperate mountain glaciers cryoconite holes mostly disappear from one melt season to the other, colonization of newly formed holes should occur. However, few studies have investigated the sources of cryoconite bacteria on temperate glaciers [22,27]. For example, on Ürümqi Glacier No. 1 (Tien Shan Mountains, China), Segawa et al. observed about half the bacterial OTUs found in the cryoconite were shared with moraines surrounding the glacier, thus suggesting that moraine can be a source of cryoconite microbes [22].

In this study, we aimed at assessing the potential sources of bacteria found in the cryoconite on the surface of Forni Glacier (Italian Alps) by investigating the similarities and the differences in bacterial community composition between cryoconite holes and the ice-marginal environments. This glacier is among the largest in the Italian Alps, is of rather easy access, and consequently has been extensively studied. Similar to other Alpine glaciers, Forni Glacier has suffered a large area reduction and the glacier tongue is retired of about 2 km in the last century [28]. During the summer season the temperature on the glacier frequently exceed +10°C and are rarely below 0°C; the snowfalls in the July-September period are increasing rare, but the rainfalls are frequent due to the thunderstorms occurrence [25]. Glaciological studies have investigated in details the mass balance of the Forni Glacier [29] and the origin and distribution of the supraglacial debris [30,31]. Ice marginal environments (i.e. glacier forefield, moraines and debris cones) are expanding on and around Forni Glacier, due to the rapid glacier shrinkage. In particular, the glacier foreland has undergone a rapid evolution for the rapid retreat of the glacier snout and the abundance of meltwater that continuously reworks the sediment. Moreover, the lateral and medial moraine have widened due to the increasing of debris availability and new debris cone have formed on the glacier surface [32]. Biological communities of the Forni Glacier area have also been investigated, particularly yeast communities in the meltwater and in the supra- and subglacial sediment [33,34] and the arthropod succession in the foreland [35]. Bacterial communities in the cryoconite holes showed temporal changes along the ablation season on the Forni Glacier, with autotrophic populations dominating communities after snowmelt, and heterotrophic populations increasing in abundance later in the season [25].

## Materials and methods

### Study area, field methods, and environmental data

**Fig 1:**
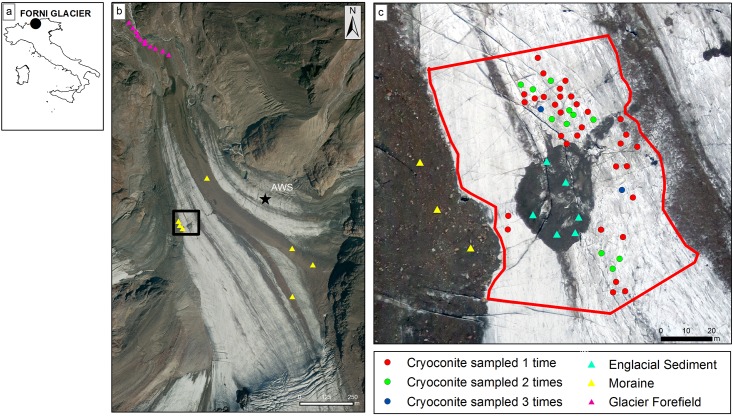
Study area. a) Position of the Forni Glacier in Italy; b) composite photograph obtained by an unmanned aerial vehicle (UAV) of the tongue of the Forni glacier with the glacier forefield. Symbols indicate the position where we collected samples on the supraglacial moraine and on the glacier forefield. The star denote the position of an automatic weather station (AWS) on the glacier surface; c) detailed UAV photograph of the study area. Position of each cryoconite hole is shown. Different symbols denote holes with overlapping positions sampled at different months. The dashed line delimits the study area.

Forni Glacier (46°12′30″ N, 10°13′50″ E; Fig 1) is one of the largest Italian valley glaciers. It covers an area of 11.34 km^2^ and ranges in elevation between 2501 and 3673 m a.s.l. [28]. The ablation season spans from early July to late September on this glacier [36] and mean monthly temperatures are above 0°C during all three months. Katabatic winds blowing from SE dominate air circulation on the glacier, but winds flowing up-valley also occur [36].

During July-October 2013, we collected 60 samples of cryoconite from cryoconite holes Twenty cryoconite samples were collected during each of three visits to the glaciers conducted on 10 July, 28 August, and 25 September 2013. In addition, on 10 July, 28 August, 25 September and 4 October, we collected seven debris samples from lateral and supraglacial moraines, six sediment samples from a large (about 30 m wide and > 3 m high) ice cored ‘dirt cone’, which occurred close to the cryoconite hole area, and ten sediment samples from the glacier forefield, which was at about 1 km from the area where we sampled the cryoconite holes. One sample of 2–5 g of cryoconite and of 30–50 g of sediment was aseptically collected from each hole or sampling location in 50 ml Falcon^™^ tubes by laboratory spoons and kept at 4°C during transport to the laboratory, which occurred within 8 h. For each sampling site, the UTM coordinates were assessed through a GPS receiver (Garmin eTrex Vista HCz, Schaffhausen, Switzerland). Full sampling details are provided in S1 Table.

Forni Glacier and its foreland are part of the Lombardy Sector of the Stelvio Park, which is managed by ERSAF Lombardia. Sampling was conducted under the framework of an agreement between the Stelvio Park-ERSAF and the University of Milan signed in 2010 and renewed every year without interruptions (principal investigator G. Diolaiuti). This study did not involve endangered or protected species.

### 16S rRNA gene fragment sequencing, sequence processing and data analysis

Total bacterial DNA was extracted from 0.7 g of cryoconite using the FastDNA Spin for Soil kit (MP Biomedicals, Solon, OH) according to the manufacturer’s instructions, and quality of extracted DNA was evaluated electrophoretically. The V5-V6 hypervariable regions of the 16S rRNA gene were PCR-amplified using 783F and 1046R primers [37,38] and sequenced by MiSeq Illumina (Illumina, Inc., San Diego, CA) with a 250 bp × 2 paired-end protocol. The multiplexed libraries were prepared using a dual PCR amplification protocol. The first PCR was performed in 3 × 75 μL volume reactions with GoTaq^®^ Green Master Mix (Promega Corporation, Madison, WI) and 1 μM of each primer and the cycling conditions were: initial denaturation at 98°C for 30 s; 20 cycles at 98°C for 10 s, 47°C for 30 s, and 72°C for 5 s and a final extension at 72°C for 2 min. The second PCR was performed in 3 × 50 μL volume reactions by using 23 μL of the purified amplicons (Wizard^®^ SV Gel and PCR Clean-up System, Promega Corporation, Madison, WI) from the first step as template and 0.2 μM of each primer. Primers contained regions complementary to the Illumina adapters and standard Nextera indexes (Illumina, Inc., San Diego, CA). The cycling conditions were: initial denaturation at 98°C for 30 s; 15 cycles at 98°C for 10 s, 62°C for 30 s, and 72°C for 6 s and a final extension at 72°C for 2 min. After the amplification, DNA quality was evaluated spectrophotometrically and DNA was quantified using Qubit^®^ (Life Technologies, Carlsbad, CA). The sequencing was carried out at Parco Tecnologico Padano (Lodi, Italy).

Forward and reverse reads were merged with perfect overlapping and quality filtered with default parameters using Uparse pipeline [39]. Suspected chimeras and singletons sequences (i.e. sequences appearing only once in the whole data set) were removed. OTUs were defined on the whole data set by clustering the sequences at > 97% of similarity and defining a representative sequence for each cluster. The taxonomic classification of the OTU representative sequences was inferred with RDP classifier [40]. Details of sequencing results for each sample are reported in S1 Table.

### Statistical methods

#### Alpha-diversity

The number of sequences at each sample varied from 2,203 to 126,734. To compare number of OTUs among samples that largely differed in the number of sequences, 2,000 reads were randomly selected from all libraries and used to calculate number of OTUs at each sample. A Generalized Linear Model (GLM) assuming a Poisson distribution and corrected for overdispersion, was used to compare the number of OTUs, which was considered an index of alpha diversity, in cryoconite and in the ice-marginal environments.

#### Beta-diversity

In order to give similar coverage to each sample while not discarding a large number of sequences from most samples, we randomly extracted 10,000 sequences from the 74 samples with a number of sequences larger than 10,000, and assessed presence or absence of each OTU on this sample of 10,000 sequences. For the remaining nine samples, presence or absence of each OTU was assessed on the original sample.

OTUs found in one sample only (singletons) were removed because they may inflate variance explained by models [41]. S1 Fig shows the number of OTUs classified at different orders.

We aimed at comparing the presence or absence of OTUs among cryoconite and ice-marginal environments. Indeed, we reasoned that the ice-marginal environments that are sources of bacteria found in cryoconite holes should share the same OTUs with cryoconite, but OTU relative abundance may differ between the two environments due to different ecological conditions. Hereafter we will refer to presence or absence of OTUs as the “composition” of a bacterial community.

This analysis was performed by Constrained Canonical Analysis (CCA) on presence or absence of OTUs [41,42]. The environment (i.e. cryoconite, moraine, dirt cone, or glacier forefield) was entered as a four-level factor. CCA was followed by post-hoc pairwise comparisons between bacterial communities at cryoconite holes on the one side, and those at each of the ice-marginal environments on the other. The rationale behind this procedure was that we were interested only in comparing bacterial community composition of cryoconite holes with those of each of the ice-marginal environments that may act as source of bacteria for them, and not in comparing composition of bacterial communities found in the different ice-marginal environments. Significance of these tests was adjusted according to the False Discovery Rate (FDR) procedure of Benjamini and Yekutiely [43]. We also checked whether significant differences detected by CCA arose because of within-habitat variation in the composition of bacterial communities [44] by performing an analysis of homogeneity of OTU composition among environments [45] with the function *betadisper* implemented in the VEGAN package [46] of R. This test is a multivariate analogue of Levene’s test for homogeneity of variance. Large dispersion within a habitat indicates that that habitat hosts heterogeneous bacterial communities.

#### Dispersal of bacteria between ice-marginal environments and cryoconite

We aimed at investigating potential dispersal of bacteria between ice-marginal environments and cryoconite. As the library sizes and the number of samples differed among environments, we first investigated rarefaction curves generated with the *rarecurve* function in VEGAN by pooling all sequences for each environment (S2 Fig). Rarefaction curves showed that a subsample of 50,000 sequences from each environment should give equal and good OTU coverage. We therefore randomly extracted 50,000 sequences from those obtained after pooling all sequence obtained from all samples collected at each environment, and assessed presence or absence of OTUs at each environment based on these samples of sequences.

We then conducted an indicator species analysis to identify taxon-habitat association patterns. This analysis was used to identify not only OTUs associated to one habitat, but also OTUs associated with two or three habitats (“indicator OTUs” hereafter). This analysis was done with the *multipatt* function (with 99,999 permutations) implemented in the INDICSPECIES package [47] of R. This procedure returns an IndVal statistics that is a measure of the strength of the association between an OTU and a habitat (or a combination of habitats) with larger numbers indicating stronger association. Also in this case, we accounted for multiple testing by correcting P-values according to the FDR procedure. Indicator OTUs with a P_FDR_ < 0.05 were considered significantly associated to a habitat or to a combination of habitats. Indicator taxa were then represented in a network by using the IGRAPH package of R, where habitats were connected by their indicator OTUs (see [27] for a similar approach). Our investigation focused on cryoconite. In order to simplify network representation, we considered only OTUs associated either with single environments or with cryoconite and one or two ice-marginal environments. That means that i.e. we represented e.g. bacteria associated to the moraine or those associated to both the moraine and the cryoconite, but not those associated to both the moraine and the dirt cone sediment, but not to the cryoconite.

All analyses were performed with R 3.1.2 [48].

## Results

### Composition of microbial communities in cryoconite and ice marginal environments

S1 Fig shows the composition of microbial communities in cryoconite and ice-marginal environments. In cryoconite, Cyanobacteria, Sphingobacteriales and Actinobacteria accounted for up to 50% of the bacterial community. Conversely, Burkholderiales and other unclassified Betaproteobacteria were the dominant taxa in ice marginal environments. Indeed, Burkholderiales abundances ranged from 15% in glacier forefield to 30% in moraine samples and the other unclassified Betaproteobacteria ranger from 13% to 20%. Cyanobacteria were virtually absent in ice-marginal environments whereas they represented up to 25% of the sequences in cryoconite. Interestingly, anaerobic Clostridiales were detected in cryoconite (5% in July), while they were less than 2% in samples from glacier forefield.

### Alpha-diversity

**Fig 2:**
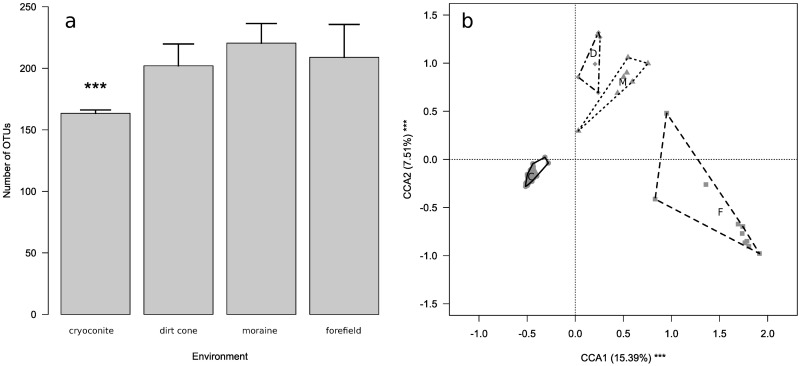
Difference in alpha and beta diversity among cryoconite holes and ice-marginal environments. a) Barplot of the number of OTUs at cryoconite holes and ice-marginal environments. Asterisks denote significant differences at post-hoc tests (*** = P < 0.001). b) Biplot of first and second components from CCA of bacterial communities in cryoconite holes, moraines, dirt cone and glacier forefield. Each symbol represents the bacterial community in one sample. Different symbols represent different environments and polygons include samples at each environment (cryoconite holes = dots and solid line, moraines = triangles and dotted line, dirt cone = diamonds and dashed-dotted line, glacier forefield = squares and dashed line,). Letters denote the centroid of bacterial communities at each environment (C = cryoconite holes, D = dirt cone, M = moraines, F = glacier forefield). The amount of variance explained by each axis is shown as well as significance of each axis as assessed by a randomization test (*** = P < 0.001).

Number of OTUs obtained from the 2,000 sequences randomly extracted per sample differed significantly among environments (F_3,79_ = 9.691, P < 0.001), being significantly lower in cryoconite holes than in ice-marginal environments, as assessed by post-hoc tests (Fig 2a).

### Beta diversity

We found significant differences in the composition of bacterial communities between cryoconite holes and ice-marginal environments (CCA: F_3,79_ = 9.301, P = 0.001; Fig 2b). Post-hoc tests also confirmed that composition of bacterial communities of cryoconite holes was different from that of all the other ice-marginal environments (F_1,15_ ≥ 12.638, P_FDR_ ≤ 0.001 in all cases). Indeed, among the 695 OTUs that occurred in the cryoconite, only 67 (14.8%) were not found in the other environments, while 674 of the 1302 (51.8%) OTUs found in any of the three ice-marginal environments we sampled were not found in the cryoconite holes (S3 Fig).

OTU heterogeneity within environments differed among environments (F_3,79_ = 98.181, P < 0.001). In particular, dispersion was lower in the cryoconite holes than in the other ice-marginal environments (Fig 2b).

### Dispersal

**Fig 3:**
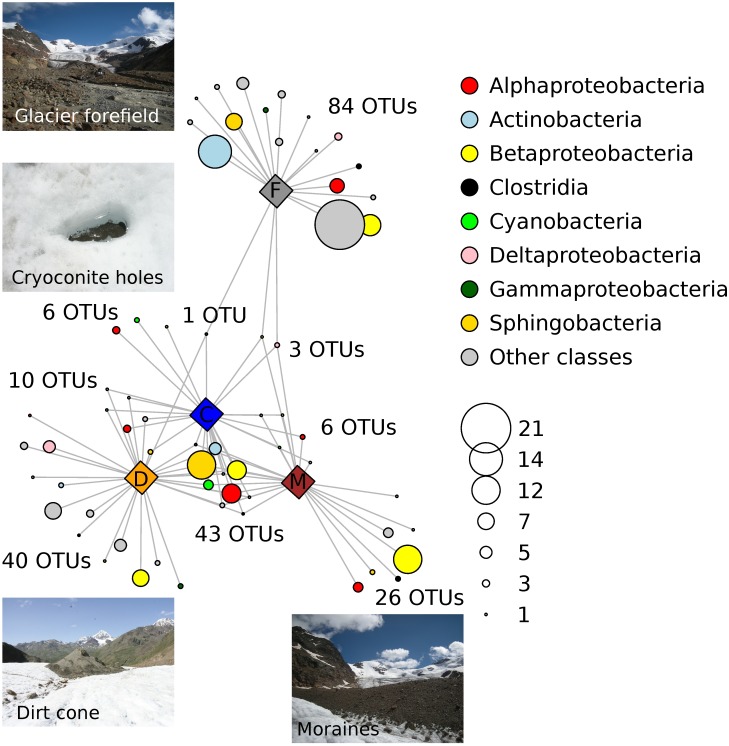
Association network. Network showing significant (P_FDR_ < 0.05) associations between indicator OTUs and specific habitats (diamonds; C = cryoconite holes, D = dirt cone, M = moraines, F = glacier forefield), or groups of habitats. OTUs (circles) were grouped according to classes. The most abundant classes are shown with different colours and circle size indicates the number of OTUs of each class (see legend in the graph). OTUs were connected to the environments to which they were significantly associated according to the Indicator Species analysis. In order to simplify the network, only OTUs associated to single habitats or to groups of habitats including the cryoconite were represented. Inserts show pictures of sampled habitats.

The indicator taxa analysis identified 219 OTUs significantly associated to one environment or to a combination of environments that included the cryoconite (S2 Table). The network analysis revealed some patterns of association among OTUs and environments. Particularly, the cryoconite holes were the environment with the lowest number of indicator OTUs. Indeed, only six OTUs were significantly associated to the cryoconite holes, while a much larger number of OTUs (63) was associated to a combination of environments including the cryoconite holes (Fig 3). Importantly, the number of OTUs significantly associated to both the cryoconite holes, the dirt cone and the moraine (43) was larger than the number of OTUs significantly associated to each of these environments. In contrast, no OTU was significantly associated to both the cryoconite and the glacier forefield. In addition, OTUs belonging to cyanobacteria were associated to the cryoconite only, or to a combination of the cryoconite, the dirt cone, and the moraine, but not to the glacier forefield.

Finally, 74 OTUs were significantly associated to both the dirt cone and the moraine, 51 to both the moraine and the glacier forefield, and 51 to all three habitats, but not to the cryoconite (these OTUs were not reported in Fig 3). No OTU was significantly associated to both the dirt cone and the glacier forefield.

## Discussion

In this study, we compared OTU composition of bacterial communities found in the cryoconite holes on the Forni Glacier in different months within the same ablation season and in three ice-marginal environments that may act as sources of bacteria found in the cryoconite. We observed large differences in the composition of microbial communities between cryoconite holes and all the ice-marginal environments we sampled (Fig 2b and S1 Fig). In addition, the cryoconite holes hosted a lower number of OTUs than these ice-marginal environments (Fig 2a).

The Hellinger distances among communities in cryoconite holes were also on average lower than those among communities in the other ice-marginal environments, as indicated by the significantly lower dispersion (Fig 2b). This indicated that communities in cryoconite holes were significantly more homogeneous than those at the other environments. Hence, not only alpha-diversity, but also beta-diversity was lower in cryoconite than in the other ice-marginal environments we investigated. These findings are in agreement with the results of previous studies on both mountain [22] and arctic glaciers [23], which showed that microbial communities in ice-marginal habitats significantly differed from those in cryoconite holes. In details, bacterial communities of ice-marginal environments showed higher biodiversity than those in cryoconite holes, suggesting that glacier surface is a more selective environment. Indeed, more than 60% of OTUs in ice-marginal samples were not present in cryoconite (S3 Fig). However composition of microbial communities appears to differ in different geographical areas, for instance different glaciers of the Greenland ice sheet [49,50]. Such differences have been attributed to different local sources of cryoconite [49]. Hence, despite hosting different bacterial communities from those of cryoconite, near-glacier environments can inoculate cryoconite holes.

Importantly, cryoconite holes showed a lower alpha- and beta-diversity, despite they were studied in larger details than the other environments. Indeed, the number of samples taken from cryoconite holes (60) was larger than the number of samples collected at all the other environments (23). Hence, the difference in sampling effort should have determined, at least, an underestimate of the total alpha- and beta-diversity of ice-marginal environments with respect to that of cryoconite holes. In addition, cryoconite samples were collected in three different months, while samples from ice-marginal environments were collected in only one time point each. However, communities in cryoconite holes were significantly more homogeneous than those of the ice-marginal environments (Fig 2). Hence, also this difference in sampling effort should have determined, at least, an underestimate of community dispersion in cryoconite holes. Finally, to reduce the possible biases arising from different coverage of environments due to differences in sampling efforts and differences in the number of sequences at each sample, we ran the analyses on a subset of 50,000 sequences randomly chosen from all the sequences from each environment. We were therefore very conservative when running our analyses. Differences in sampling effort among environments should therefore have not affected our conclusions.

The indicator species analysis and the network analysis indicated that 59 OTUs were significantly associated to a combination of environments including the cryoconite holes, the dirt cone, and the moraines. In contrast, we found that four OTUs only were significantly associated to both the cryoconite holes and the glacier forefield, while a larger number of OTUs was also associated to either the moraine or the dirt cone sediments (Fig 3). These results suggest that the dirt cone and the moraine sediments were important sources of bacteria for the cryoconite. These ice-marginal environments are very close to the cryoconite holes we sampled (20–150 m), while the glacier forefield is more distant (800–1,000 m) and 200 m lower. Despite katabatic winds dominating air circulation on the Forni glacier, periodic winds flowing up-valley are not rare, and may therefore transport sediment and bacteria from the glacier forefield [36,30]. However, our results suggest that aeolian transport of sediment that forms the cryoconite, and of the associated bacteria, may occur more easily from surrounding environments than from those down-valley [13,50].

This pattern is consistent with an assembly of bacterial communities of cryoconite holes due to species sorting [51]. Indeed, environmental conditions of cryconite holes strongly differ from those of the ice-marginal environments for temperature, solar irradiation and the presence of water that may favour typical freshwater inhabitants. Without dispersal limitation between ice-marginal environments and glacier surface, the community composition of cryoconite holes is determined by the different environmental conditions between them and the ice-marginal environments, which allow recruiting into the community only those taxa that in the cryoconite holes find the conditions to outcompete the other populations. For example, Cyanobacteria are the most abundant taxon in cryoconite holes and among the less abundant ones in ice-marginal environments (S1 Fig). Moreover, as previously suggested, dispersal and species sorting processes might not be unidirectional. Indeed, the wash-out of cryoconite holes due to ablation and the consequent transport of bacterial communities could inoculate downstream locations with microorganisms [10,52–54].

Interestingly, we observed that, despite cryocontite holes were aerobic environments [24], anaerobic Clostridiales (phylum Firmicutes) were more abundant in cryoconite than in other samples (S1 Fig). Clostridiales have already been recorded in cryconite from Tyrolean Alps [15] and from Antarctica [55]. and they dominated bacteria collected in snow and dust traps on the Greenland Ice Sheet [56]. Unfortunately, in this study, we did not collect snow or dust samples deposed on the glacier surface by long-range air transport. This may have limited our ability to assess the importance of long-range transports in seeding bacterial communities of cryoconite. However, we consider unlikely that snow may provide inocula for the bacterial communities in the cryoconite holes of Forni Glacier. Indeed, snow samples collected on Forni Glacier in spring 2014 (i.e. some months after we sampled cryoconite) were dominated by Burkholderiales and Cytophagales (I. Tagliaferri unpublished data) and strongly differed from those observed in the cryoconite. Despite bacterial communities in different snowfalls may differ, the same taxa dominated snow samples collected in different areas of the world (Alps, Anatolia, Karakoram and Himalaya) in different years (I. Tagliaferri unpublished data), thus suggesting that bacterial communities in the snow are not an important source of bacteria for cryoconite holes.

In summary, the results reported in the present study indicate that cryoconite holes host different and less diverse bacterial communities than ice-marginal environments. However, taxa are probably recruited from the surrounding environments and the bacterial populations not adapted to this supraglacial habitat are filtered out. Our results therefore support the hypothesis that species sorting processes mainly drive the composition of bacterial communities of the cryoconite holes.

## Supporting information

S1 Table Geographic location, number of sequences and number of OTUs of samples. Cryoconite July: n = 20; Cryoconite August: n = 20; Cryoconite September: n = 20; Dirt cone: n = 6; Moraines n = 7; Glacier forefield: n = 10. Asterisks denote samples whose relative abundance of OTUs was normalized to 10,000 in the analyses of beta-diversity.
(XLSX)

S2 Table Results from indicator species analysis aiming at identifying OTUs typical of different environments or groups of environments. The classification of OTUs at class and order level (if available) is reported, as well as the value of the IndVal statistic and the significance of the test, corrected with the False Discovery Rate procedure.
(XLSX)

S1 Fig Microbial community structures. The mean relative abundance of bacterial orders (with the only exception of Cyanobacteria that were grouped at class level) in different samples is reported.
(TIF)

S2 Fig Rarefaction curves generated by pooling all sequences for each environment. The vertical dashed line indicates 50,000 sequences.
(TIF)

S3 Fig Venn diagram showing the number of OTUs shared by cryoconite holes and ice-marginal environments. (TIF)

S1 File All data used for the analyses. Details on all variables are included separate sheets within the file.
(XLSX)
