# Supplementary figures and images for: Potential sources of bacteria colonizing the cryoconite of an Alpine glacier
Source: PLoS One. 2017 Mar 30;12(3):e0174786. doi: 10.1371/journal.pone.0174786 (PMC5373619; doi:10.1371/journal.pone.0174786)

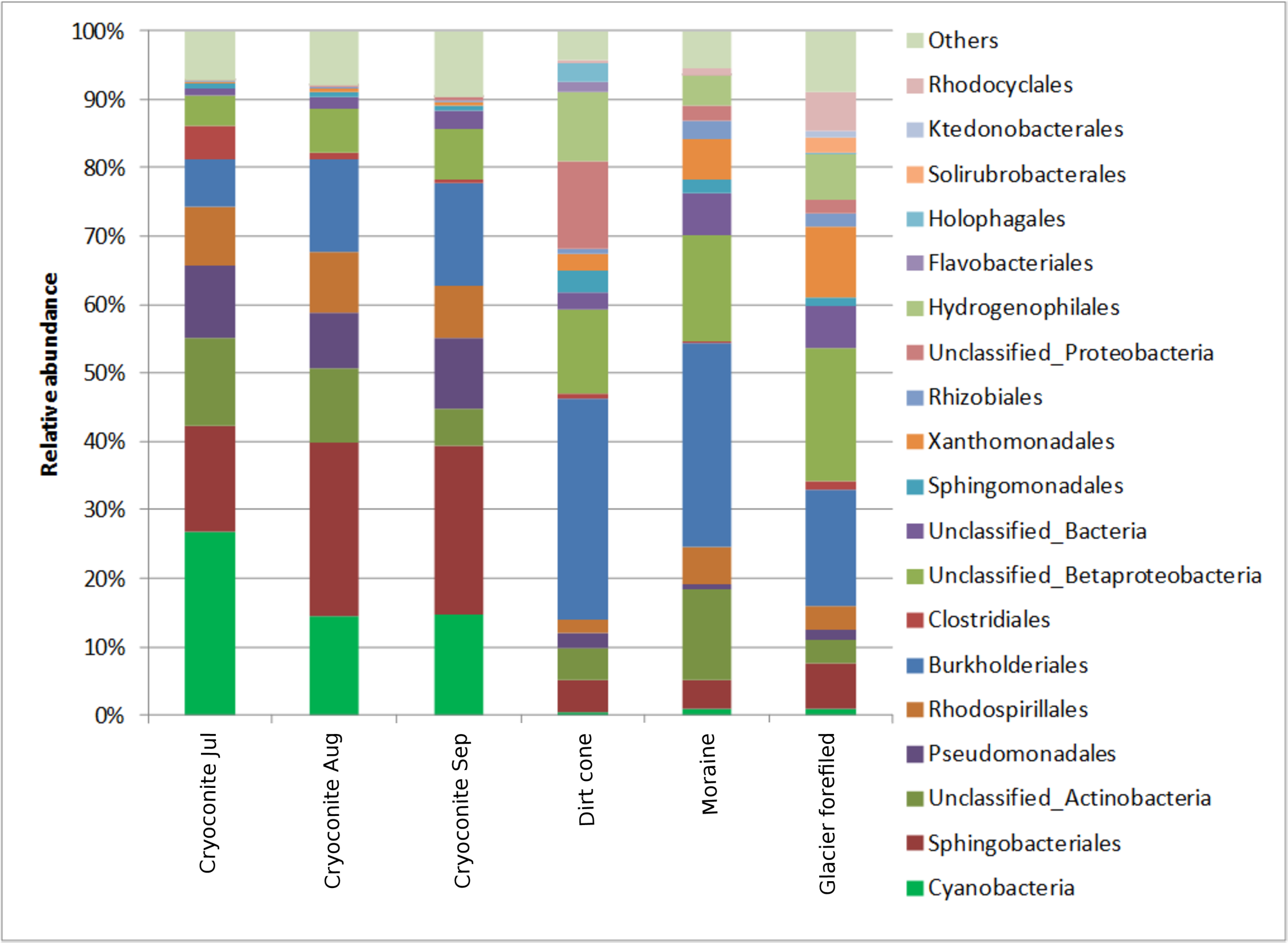

Supplement: S1 Fig — The mean relative abundance of bacterial orders (with the only exception of Cyanobacteria that were grouped at class level) in different samples is reported. (TIF) [file pone.0174786.s003.tif]

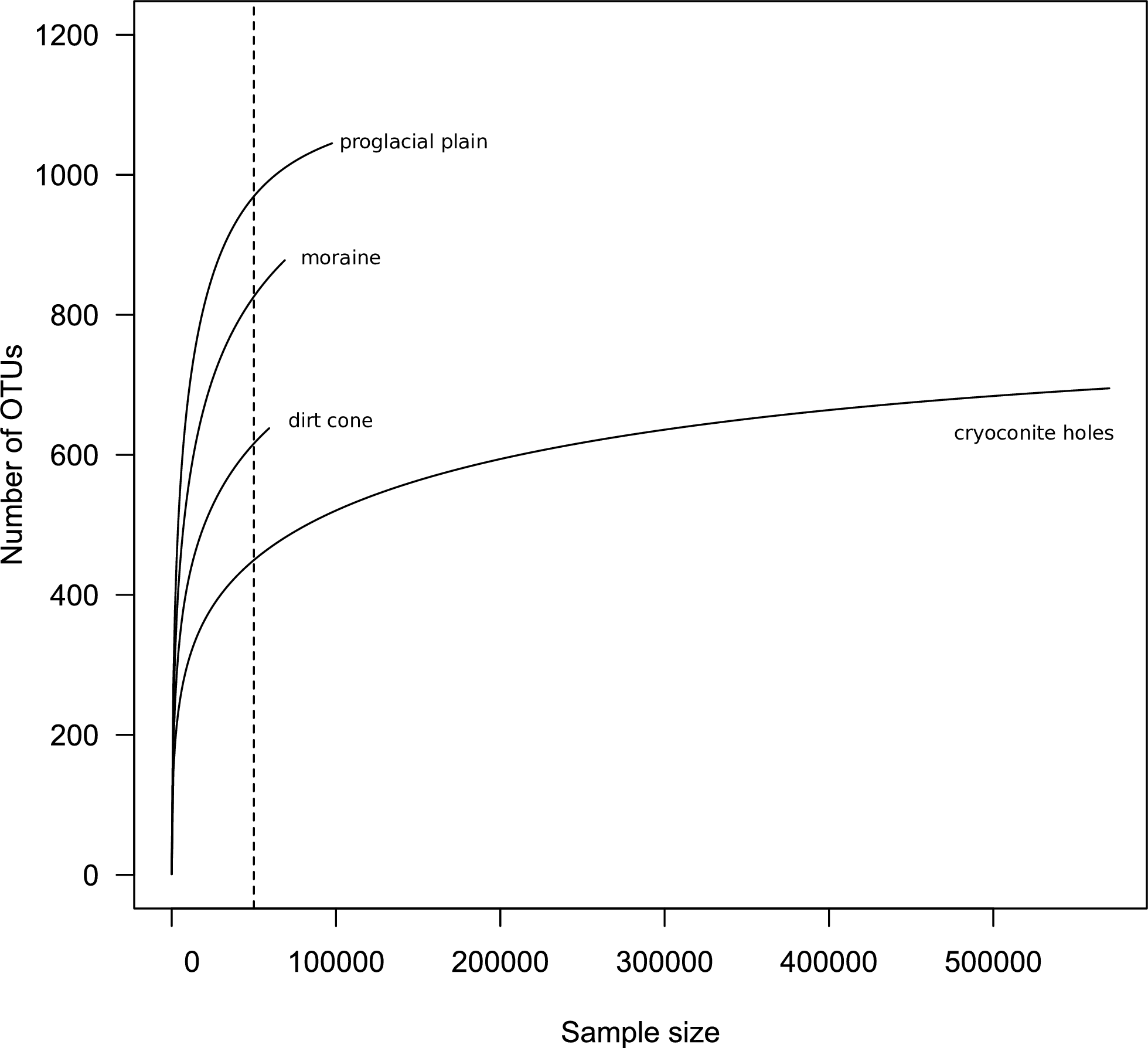

Supplement: S2 Fig — The vertical dashed line indicates 50,000 sequences. (TIF) [file pone.0174786.s004.tif]

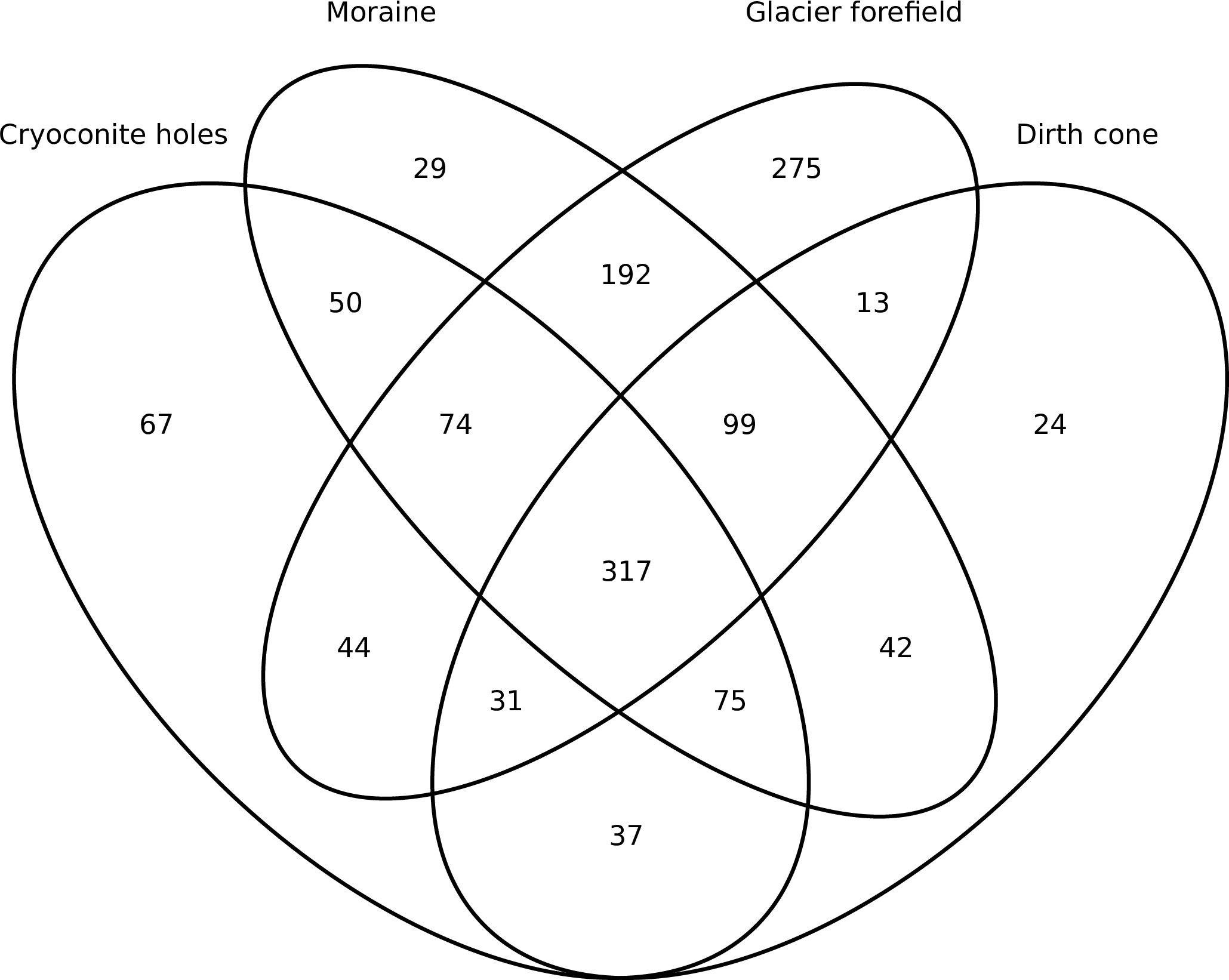

Supplement: S3 Fig — (TIF) [file pone.0174786.s005.tif]
